# Amino Acid Composition of Thirty Food Fishes of the Ganga Riverine Environment for Addressing Amino Acid Requirement through Fish Supplementation

**DOI:** 10.3390/foods13132124

**Published:** 2024-07-03

**Authors:** Basanta Kumar Das, Satabdi Ganguly, Supriti Bayen, Anjon Kumar Talukder, Archisman Ray, Subhadeep Das Gupta, Kajal Kumari

**Affiliations:** ICAR-Central Inland Fisheries Research Institute, Kolkata 700120, India

**Keywords:** amino acids, fish, Ganga river, HPLC, protein, fish supplementation

## Abstract

Amino acids are significant biomolecules that govern the major metabolic processes and act as precursors for macromolecules such as proteins that are crucial to life. Fish is an integral component of human nutrition and a dietary source of high-quality animal proteins and amino acids. In this context, the crude protein and amino acid compositions of food fish from different landing stations of the Ganga river have been determined. The Kjeldahl method was utilized to determine the crude protein content and the amino acids were analyzed using high-performance liquid chromatography (HPLC); data on 30 food fish were assessed. The study showed that among the fish studied, *Eleotris fusca*, *Macrobrachium malcomsonii*, and *Mystus cavasius* were rich in most of the amino acids important for human nutrition, such as glycine, glutamic acid, cysteine, threonine, phenylalanine, methionine, lysine, leucine, isoleucine, histidine, and valine. Further, it was observed that the daily consumption of these fish (approximately 50 g) can fulfil the daily requirement of these individual amino acids for an adult human with a body weight of 60 kg. Therefore, the amino acid composition analyzed in the present study could be utilized for recommendation by clinicians according to the requirement for specific amino acids, and fish can be prescribed as a natural supplement against the amino acid requirement.

## 1. Introduction

Food insecurity is a multidimensional phenomenon characterized by the non-availability of food due to famine and hunger. Protein hunger mainly indicates a dietary protein deficiency in the daily diet required to meet our body’s needs. Protein hunger and protein–calorie malnutrition (PCM) or protein–energy malnutrition (PEM) are major public health issues that mainly occur due to an inadequate intake of protein and energy, respectively [1]. Proteins are essential components of the human diet that perform several important biological functions and are the major structural component of muscle and other tissues. Amino acids (AAs) are considered the building blocks of protein in cellular mechanisms and act as intermediates in the pathway of metabolic health. Protein and protein complexes are essential for vitality [2], as they are broken down into AAs, which can be absorbed and utilized by the body. In addition, the important role of AAs has also been shown as a regulator of gene expression [3]; however, unlike fats (lipid form in the adipose tissue) and carbohydrates (glycogen form in the muscle and liver), there is no dedicated storehouse in the body for proteins [4]. Several preclinical studies have proven that metabolic health can be promoted through the restriction of dietary protein [5,6,7,8], and the quality and characteristics of dietary protein are evaluated from the ratio of essential amino acid (EAA) to nonessential amino acid (NEAA) [9]. Branched-chain amino acids (BCAAs) are not only considered a major supplier of dietary protein for metabolic health [10], but also there is a correlation between the protein intake and the circulation of BCAA levels [11]. High-quality proteins contain dietary EAAs in a digestible form that might be compatible with the human health requirements [12,13]. An insufficient uptake of protein and calories in the diet can trigger malnutrition and ultimately lead to hidden hunger. Since the 1990s, the primary focus in developing countries has been on preventing specific micronutrient deficiencies, such as PCM, in particularly vulnerable age groups. Of the approximately 159 million stunted children worldwide, 36 nations were found to account for 90% of the total number of stunted children. Of these children, 74 million were believed to be in South Central Asia [14]. The physical growth and mental development of infants and school children can be affected due to malnutrition, whereas in adults, productivity and work performance, as well as a woman’s reproductive system, can be negatively impacted [15]. Therefore, finding the cheapest source of quality protein will always be a priority for nutritionists and researchers. Fish is often well-known as an important source of the cheapest animal proteins, as there are many different types of fish and their prices differ greatly. As a result, it is available to all socioeconomic classes, including those with high, medium, and low incomes, making it one of the most widely consumed animal protein sources. Fish proteins in the form of EAAs play a crucial role in fulfilling the human nutritional requirements, as the body cannot synthesize the nine EAAs required to be supplied through the diet to make proteins. The inclusion of nutrient-dense small indigenous fish (SIFs) in a regular diet might be a good strategy to utilize the animal protein and nutrients. The SIFs are essential to the rural communities of India, particularly the fishermen whose livelihoods rely heavily on the catch of these SIFs. Although recorded in the catch data, they are kept aside for consumption, which unknowingly helps them to meet their daily protein needs. Therefore, priority should be given to generating nutritional information on the several SIFs that are abundant in inland open waters, such as rivers, lakes, wetlands, and other derelict water bodies. The present study was carried out to assemble information on the amino acid composition and protein content of commercially important food fish, especially the SIFs, with an approach towards enhancing the opportunity of the proper utilization of fish for the dietary requirements in human health and nutrition from the river Ganga, which harbors more than 190 fish species [16] and provides nutrition to a large population of India. The study had a scientific approach to amino acid types that would meet the requirement for hidden hunger in the Asian and African countries of the rural population.

## 2. Materials and Methods

### 2.1. Ethical Statement

The study and fish sample collection, experimental procedures, and fish sacrifice followed the study country’s ethical guidelines, i.e., India, and as per the Institute Animal Ethics Committee (IAEC) (Number: CIFRI/IAEC-21-22/01).

### 2.2. Study Area

**Figure 1:**
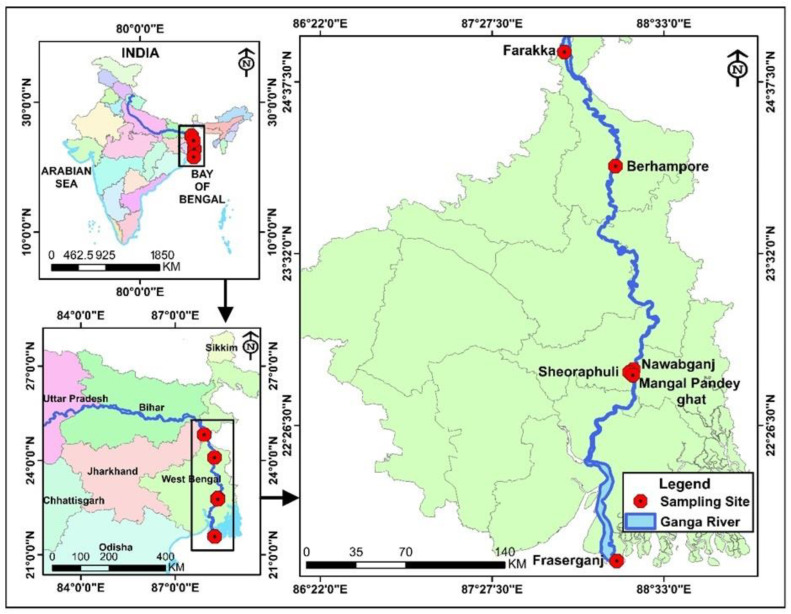
Sampling sites of the fish sample collection. The black boxes in the figure represent the sampling sites on the river Ganga.

The field sampling was conducted along the Ganga river in different sections to collect the fish samples. The sampling sites covered the districts of Murshidabad, South 24 Parganas, and North 24 Parganas in the state of West Bengal, India. A total of six stations were selected based on the availability of the fish species along the river Ganga, namely Farakka, Behrampur, Nawabganj, Sheoraphully, Mangal Pandey ghat, and Fraserganj. The main river channels and adjoining landing centers were visited for the fish sample collection. The surveyed sampling sites are given in Figure 1.

### 2.3. Fish Sample Collection and Identification

During the collection of the fish samples, different selective and non-selective fishing gears, viz., seine nets, gill nets, barrier and falling nets, cast nets, bag nets, drag nets, traps, etc., were used. The fish were identified according to their taxonomic characteristics, as described by Talwar and Jhingran [17], Nelson [18], and Jayaram [19].

### 2.4. Sample Processing

The freshly caught fish from the river Ganga at different sites were collected, kept in ice, and brought to the laboratory. In total, 30 fish species were included in the crude protein content and amino acid profiling: *Rhinomugil corsula*, *Glossogobius giuris*, *Eleotris fusca*, *Salmostoma bacaila*, *Setipinna phasa*, *Apocryptes bato*, *Clupisoma garua*, *Lepidocephalichthys guntea*, *Cynoglossus cynoglossus*, *Macrognathus pancalus*, *Gagata cenia*, *Gonialosa manmina*, *Chanda nama*, *Parambassis ranga*, *Nandus nandus*, *Cirrhinus reba*, *Cabdio morar*, *Crossocheilus latius*, *Eutropiichthys vacha*, *Silonia silondia*, *Secutor ruconius*, *Macrobrachium malcolmsonii*, *Channa marulius*, *Acanthocobitis botia*, *Mystus cavasius*, *Anodontostoma chacunda*, *Coilia dussumieri*, *Corica soborna*, *Securicula gora,* and *Glyptothorax telchitta*. The collected fish were first segregated according to size, i.e., large fish and SIFs. The SIFs are fish that attain a maximum length of 20 cm at maturity or the adult stage. Therefore, according to this classification, all were SIFs except for *Eutropiichthys vacha* and *Silonia silondia* (large fish), and *Macrobrachium malcolmsonii* (shellfish). The large fish (*n* = 6) were descaled and deboned, and the fillets were homogenized. The SIFs were cleaned, descaled, and degutted, and the edible sections were gathered (the edible portions in the SIFs included all the bones and the heads). Six pooled samples were prepared for each SIF (*n* = 50). All the fish samples were then stored at −40 °C until further processing.

### 2.5. Analysis of Amino Acids

The Kjeldahl technique was used for the crude protein estimation [20]. The following formula was used for the calculation of the crude protein content.

Protein content (%) = (X × 0.14 × V × 6.25* × 100)/(1000 × V1 × W)

where,

X = Titer value

V—Total volume of digest

V1—Volume of the digest for distillation

W—Weight of sample for digestion

1 mL 0.01 N N/100 H2SO4~0.00014 g nitrogen or (0.14/1000)

* The nitrogen content of most fish/meat protein is 16%. Hence, 1 g nitrogen equivalent of protein is 100/16 or 6.25.

Briefly, for the amino acid analysis, the fish samples (50 mg) were hydrolyzed with 6N hydrochloric acid (5 mL) under anaerobic conditions at 110 °C for 12 h and then neutralized with 6N NaOH [21]. The neutralized samples were then derivatized using an AccQ-Fluor Reagent kit (WAT052880, Waters, Milford, MA, USA). The samples, after derivatization, were loaded into a C18 RP column- and fluorescence detector-equipped high-performance liquid chromatography (1525, Waters) system (2475, Waters). The individual AAs (17 in total) were identified and measured by comparing them with the standards (WAT088122, Waters), except for tryptophan.

### 2.6. Statistical Analysis

The means and standard deviations were analyzed in MS Excel, 2010.

### 2.7. Daily Value (DV%) and Potential Contribution to Recommended Dietary Allowance (RDA)

The potential contribution of the studied food fish from the river Ganga to the DV% of the food was calculated from the RDA for an adult man with a body weight of 60 kg [22].

## 3. Results and Discussion

AAs are prerequisites for protein synthesis and other important nitrogen-containing compounds in the body; however, the proportions may vary as per the characteristics of a particular protein. The EAAs required for protein synthesis must be supplied through the diet; therefore, the amino acid composition of the food proteins determines their nutritional quality. In this context, amino acid profiling can give comprehensive information about any food commodity’s amino acid composition. AAs are considered the primary nutritional factor of dietary protein to synthesize the tissue and organ proteins for normal growth and function in the human body [23,24]. The classification of AAs is often categorized according to nutritional basis, as EAAs and NEAAs or conditionally essential amino acids (CEAAs) [3]. Moreover, AAs are concerned with health problems and deficiencies in AAs can lead to several diseases. AAs such as arginine, histidine, cystine, lysine, leucine, threonine, methionine, tyrosine, valine, and tryptophan are the EAAs. In contrast, glutamine, glycine, glutamic acid, proline, and taurine are the CEAAs, and aspartic acid, alanine, and serine are the NEAAs for humans [9]. However, the functional amino acids (FAAs) have been proposed in recent years, and the participation of FAAs is considered for regulating the key metabolic pathways concerning the improvement of health, survival, development, growth, lactation, and reproduction, as well as the prevention of diseases [3,25]. AAs are also classified according to the taste rendered by them, like the sweet taste AAs (serine, alanine, and glycine), and the umami taste AAs (aspartic acid and glutamic acid) [26].

Fish can contribute significantly as a rich source of animal proteins, and their easy digestibility and higher satiety effect compared with other animal protein sources make them an indispensable choice for formulating a balanced diet [27]. In the present study, the amino acid composition and protein content of 30 selected food fish from the Indian subcontinent were analyzed (Table 1), which could be useful in clinical practices, and particular fish species could be recommended in the diet to gain better nutritional aspects. There was no significant variation in the AA content of the fish belonging to the same species from the different landing stations of the Ganges. The river Ganges is a rich hub of freshwater fish diversity; hence, a wide variety of options are available for taking the animal protein to consumers in an affordable range. The fish species rich in specific AAs are listed in Table 2.

Arginine is a precursor for nitric oxide synthesis and is vital for neurotransmission, blood clotting, and blood pressure maintenance. Cell division, the removal of ammonia, wound healing, immune function, and hormone regulation are critically controlled by arginine. It has great significance in enhancing the rate of recovery for several diseases, such as sepsis, hypertension, preeclampsia, anxiety, erectile dysfunction, and many others. *Eleotris fusca* (4.90 ± 0.36 g/100 g) was found to be rich in arginine, followed by *Securicula gora* (3.17 ± 0.34 g/100 g) and *Mystus cavasius* (2.28 ± 0.18 g/100 g). Shahidi et al. [28] reported approximately 5% arginine in the small forage fish, capelin (*Mallotus villosus*). High arginine in coldwater fish, such as *O. mykiss*, *T. putitora*, and *N. hexagonolepis*, was reported by Mohanty et al. [9]. These arginine-rich SIFs could be recommended for arginine deficiency, where fish are available in warmer climates.

Leucine performs a unique role in the human metabolism [29] and has remedial effects in severe injurious conditions, such as burn, sepsis, and trauma [30]. Several freshwater fish were found to be rich in leucine, viz. *Eleotris fusca*, *Mystus cavasius*, *Silonia silondia*, and *Cynoglossus cynoglossus*; however, the leucine content was also high in the shellfish *Macrobrachium malcolmsonii* (3.08 ± 0.32 g/100 g). A high level of leucine in the molting stage of *M. malcolmsonii* has been documented in recent years [31]. Marine fish were reported to have high levels of leucine [32], though carp and catfish in the freshwater zones were found to have higher levels of leucine content than the marine fish [9].

Methionine is the only sulfur-containing EAA that is a precursor for other AAs [33,34]. Methionine helps in the buildup of nails and boosts the flexibility and nature of the skin as well as the hair [35]. Methionine has a great role in the prevention of liver damage due to acetaminophen poisoning [36]. Other uses are reported as healing asthma, Parkinson’s disease, radiation side effects, allergies, depression, schizophrenia, and drugs and alcoholism [37]. The methionine-rich fish were found to be *Eleotris fusca* (1.92 ± 0.18 g/100 g) followed by *Securicula gora* (1.45 ± 0.11 g/100 g) and the shellfish *Macrobrachium malcolmsonii* (1.26 ± 0.12 g/100 g) among the fish and prawns analyzed in the present study. Methionine is reported in high amounts in animal meat, including marine fish such as mahi-mahi, halibut, and salmon [38]; even another marine fish, *S. waitei*, and the coldwater fish, *T. putitora*, were found to be the highest among the fish, and also higher than mutton [39].

Glutamate is critical in human nutrition, metabolism, signaling, and protein structure [40]. *E. fusca* (7.61 ± 0.63 g/100 g) was found to be the highest in glutamic acid among the studied fish and the other fish rich in glutamic acid were *Mystus cavasius*, *Acanthocobitis botia*, and *Cynoglossus cynoglossus*. Carps such as *L. rohita*, *C. catla*, and *C. mrigala* and catfish such as *C. magur* and *H. fossilis* were found to be rich in glutamic acid [9] and other fish species such as mackerel [41], as well as red salmon [42].

Glycine plays a vital role in metabolism, enhancing anti-antioxidant activity and wound healing, promoting protein synthesis, preventing tissue injury, and improving immunity and other treatments as metabolic disorders in cardiovascular disease, obesity, diabetes, cancer, and ischemia-reperfusion injuries, as well as various inflammatory diseases [43]. *Eleotris fusca* was found to have the highest glycine (3.10 ± 0.28 g/100 g) followed by the shellfish *Macrobrachium malcolmsonii* (2.53 ± 0.14 g/100 g) in the present study. Previously, the glycine content of the European seabass, turbot, gilthead seabream, *Channa micropeltes*, *Channa striatus*, and *Channa lucius* was reported to be high [32,44].

Histidine has multiple functions in protein interaction [45] and is also a precursor of histamine. It is also essential for tissue health, the maintenance of the myelin sheaths, and the elimination of heavy metals from the body [46]. *Eleotris fusca* (1.77 ± 0.021 g/100 g) contained the highest histidine level among the studied fish. Previous studies have recommended that small indigenous fish such as *A. testudineus*, *A. mola*, and *P. sophore* are good histidine sources [47].

Lysine is considered one of the important EAAs for optimal growth, and immunodeficiency can be developed due to its deficiency [48]. It has clinical use in preventing and treating cold sores or fever blisters [9]. *Eleotris fusca* (4.06 ± 0.33 g/100 g) was found to have the highest level of lysine in the present study; however, a previous study reported high lysine levels in *Channa* spp. [49].

Different types of nervous system disorders, including spinal spasticity, familial spastic paraparesis, multiple sclerosis, and amyotrophic lateral sclerosis, are clinically treated using threonine [50]. The present study found the highest level of threonine in *S. gora* (2.69 ± 0.21 g/100 g) fish species. Fish can be recommended for threonine as a natural supplement [9].

Isoleucine is a branched-chain EAA required to build muscle, recover muscle fatigue, and relieve muscle soreness. *Eleotris fusca* (3.10 ± 0.24 g/100 g) was found to have the highest level of isoleucine in the present study. Coldwater fish such as *Oncorhynchus mykiss* were reported to have a high content of isoleucine by Mohanty et al. [9].

**Figure 2:**
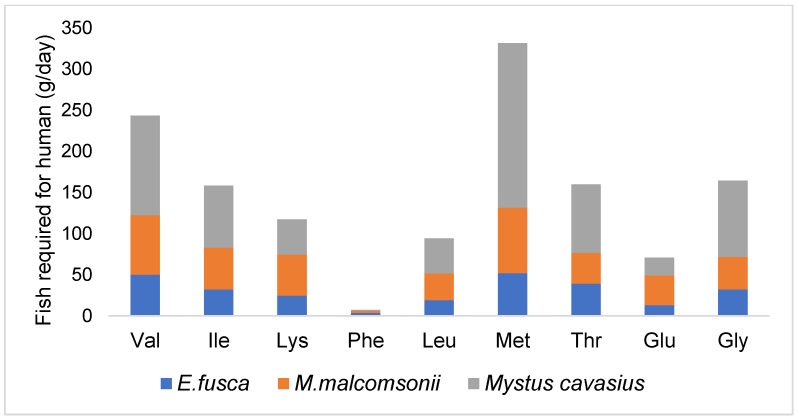
Fish required for human (g/day) consumption for fulfilling the amino acid requirement of an adult human weighing 60 kg, as per Joint FAO/WHO/UNU, 2007 [12].

NEAAs have significant roles in the human body, such as gene expression, tumor metabolism, cell signaling, blood flow, the transportation of nutrients and metabolism, and the development of brown adipose tissue. Other important regulated factors include intestinal microbial growth, metabolism, anti-oxidative responses, and immune responses [3,9]. The SIF, *E. fusca*, along with the shellfish, *M. malcomsonii*, and the catfish, *M. cavasius*, was also found to be rich in NEAAs such as glutamic acid, glycine, alanine, aspartic acid, and serine. Therefore, the consumption of these fish can fulfil the daily requirement of most of the AAs (both EAAs and NEAAs) for human nutrition according to the requirements of an adult human weighing 60 kg [12] (Figure 2).

**Figure 3:**
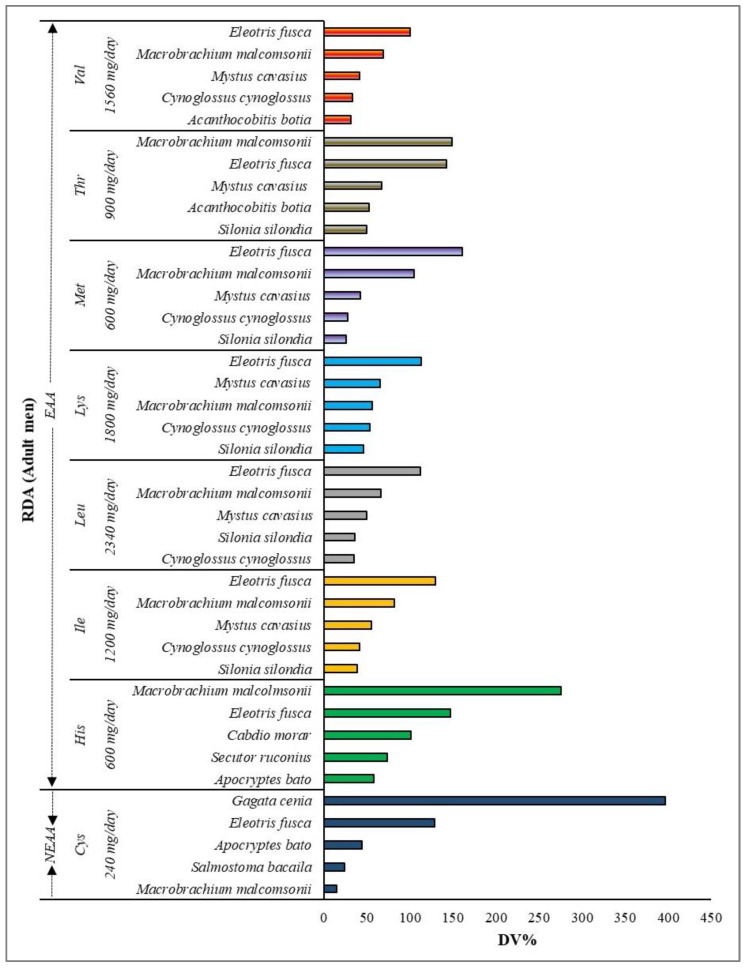
The RDA for adult men (60 kg weight) is plotted along the *Y*-axis, and the potential contribution of the fish studied (top five) (DV%) is presented along the *X*-axis. DV% was calculated using the determined amino acid concentration and the RDA following the Joint FAO/WHO/UNU guidelines (2007) [12]. For calculating the DV%, each serving was considered as 50 g.

The RDA provides a quantitative nutrient recommendation for maintaining general health in the population. For instance, this might not be the same for children, adult males, or adult females. With minor changes, the Indian Council for Medical Research (ICMR) supports drafting the RDA guidelines following the joint FAO/WHO/UNU guidelines [12]. A nutrient’s RDA will be expressed in a standard unit, making it easier for the average person to determine their needs based on their individual body weight and/or basal metabolic rate. The potential contribution of the studied top five fish, rich in the respective AAs (DV%), to the RDA for adult men with a 60 kg weight (valine, threonine, methionine, lysine, leucine, isoleucine, histidine, and cysteine) are depicted in Figure 3. The richness in the specific AAs of the studied fish (Table 2) is based on its contribution (DV%) to the RDA for that particular amino acid; for example, the SIF, *E. fusca*, was found to be very rich in methionine contributes (50 g serving), approximately 160% of the daily methionine requirement.

## 4. Conclusions

The Ganga river system nurtures a vast biodiversity of fish, an important food and nutrition source. The inclusion of nutrient-dense indigenous fish in a regular diet can be a good strategy to utilize the cheapest animal protein and nutrients. Amino acid-rich food fish may be suitable for both consumers and clinical trials. In the present study, indigenous fish of the Ganges, such as *E. fusca*, *M. malcomsonii*, and *M. cavasius* can fulfil the requirement of AAs such as glycine, glutamic acid, cysteine, threonine, methionine, phenylalanine, lysine, isoleucine, leucine, histidine, and valine. Therefore, specific fish species could be recommended based on the amino acid requirements to patients or consumers. Further studies and research on the nutrient benefits of indigenous fish species might develop a scientific approach in clinical aspects.

## Figures and Tables

**Table 1:** Amino acid composition and protein content of 30 food fish from the river Ganga (g/100 g).

Amino Acid (g/100 g)	*L. guntea*	*S. ruconius*	*G. cenia*	*C. morar*	*M. malcomsonii* ^†^	*R. corsola*		Requirement for Adult Human (g/kg body wt./day) (WHO, 2007) [12]
**Crude protein (%)**	15.8 ± 0.01	13.7 ± 0.5	13.5 ± 0.11	15.6 ± 0.04	15.5 ± 0.09	17.3 ± 0.24		
Essential amino acids (EAAs)								
ARG	1.10 ± 0.14	0.35 ± 0.01	0.16 ± 0.01	1.62 ± 0.14	0.35 ± 0.01	0.09 ± 0.0		-
VAL	0.46 ± 0.02	0.59 ± 0.02	0.51 ± 0.04	0.84 ± 0.09	2.15 ± 0.19	0.51 ± 0.4		0.026
HIS	0.19 ± 0.01	0.89 ± 0.06	0.23 ± 0.01	1.21 ± 0.11	3.30 ± 0.32	0.28 ± 0.02		0.01
ILE	0.43 ± 0.01	0.51 ± 0.03	0.49 ± 0.02	0.41 ± 0.02	1.97 ± 0.21	0.46 ± 0.06		0.02
LEU	0.71 ± 0.04	0.92 ± 0.06	0.80 ± 0.06	0.78 ± 0.12	3.08 ± 0.32	0.85 ± 0.12		0.039
LYS	0.95 ± 0.03	0.97 ± 0.05	0.91 ± 0.08	0.93 ± 0.06	2.01 ± 0.19	0.92 ± 0.11		0.030
MET	0.02 ± 0.00	0.11 ± 0.01	0.04 ± 0.0	0.08 ± 0.0	1.26 ± 0.12	0.16 ± 0.01		0.010
PHE	0.40 ± 0.01	0.49 ± 0.02	0.46 ± 0.02	0.40 ± 0.01	2.51 ± 0.19	0.45 ± 0.06		Tryo + phyla = 0.025
THR	0.47 ± 0.05	0.71 ± 0.03	0.45 ± 0.04	0.06 ± 0.0	2.67 ± 0.24	0.58 ± 0.09		0.015
Nonessential amino acids (NEAAs)								
CYS	-	-	1.90 ± 0.11	-	0.07 ± 0.0	-		0.004
GLU	1.91 ± 0.21	0.82 ± 0.05	1.83 ± 0.14	1.16 ± 0.12	2.85 ± 0.22	1.62 ± 0.14		-
GLY	1.01 ± 0.11	0.85 ± 0.06	0.48 ± 0.02	0.11 ± 0.01	2.53 ± 0.14	0.34 ± 0.02		-
PRO	0.55 ± 0.02	0.47 ± 0.01	0.36 ± 0.02	0.39 ± 0.01	0.08 ± 0.0	0.38 ± 0.06		-
TYR	0.29 ± 0.01	0.35 ± 0.01	nd	nd	8.06 ± 1.14	0.38 ± 0.01		Tryo + phyla = 0.025
ALA	0.74 ± 0.06	0.70 ± 0.04	0.59 ± 0.03	0.59 ± 0.03	2.02 ± 0.18	0.65 ± 0.11		-
ASP	1.16 ± 0.12	0.09 ± 0.01	1.14 ± 0.20	0.53 ± 0.02	0.10 ± 0.01	0.88 ± 0.09		-
SER	0.51 ± 0.05	0.71 ± 0.04	0.47 ± 0.02	1.08 ± 0.09	2.17 ± 0.22	0.56 ± 0.08		-
**Amino Acid** **(g/100 g)**	***C. marulius***	***A. bato***	***M. panculus***	***C. cynoglossus***	***S. phasa***	***P. ranga***		**Requirement for Adult Human (g/kg body wt./day)** [12]
**Crude protein (%)**	17 ± 0.33	16.95 ± 0.08	18.1 ± 0.03	17.1 ± 0.12	22.2 ± 0.04	11.7 ± 0.21		
Essential amino acids (EAAs)								
ARG	0.99 ± 0.22	0.07 ± 0.00	1.94 ± 0.25	1.80 ± 0.12	0.69 ± 0.06	0.91 ± 0.07		-
VAL	0.47 ± 0.03	0.40 ± 0.03	0.88 ± 0.09	1.04 ± 0.08	0.35 ± 0.02	0.38 ± 0.02		0.026
HIS	0.24 ± 0.01	0.70 ± 0.08	0.45 ± 0.04	0.51 ± 0.04	0.17 ± 0.01	0.18 ± 0.01		0.01
ILE	0.42 ± 0.03	0.02 ± 0	0.84 ± 0.10	0.99 ± 0.08	0.29 ± 0.01	0.34 ± 0.01		0.02
LEU	0.75 ± 0.08	0.73 ± 0.08	1.45 ± 0.14	1.65 ± 0.14	0.58 ± 0.03	0.66 ± 0.03		0.039
LYS	0.74 ± 0.09	0.93 ± 0.09	1.46 ± 0.11	1.92 ± 0.21	0.66 ± 0.04	0.32 ± 0.01		0.030
MET	0.04 ± 0.00	0.12 ± 0.01	0.25 ± 0.02	0.33 ± 0.01	0.14 ± 0.01	0.14 ± 0.11		0.010
PHE	0.42 ± 0.03	0.38 ± 0.04	0.79 ± 0.08	0.90 ± 0.09	0.31 ± 0.01	0.34 ± 0.01		Tryo + phyla = 0.025
THR	0.42 ± 0.01	0.87 ± 0.10	0.83 ± 0.10	0.86 ± 0.10	0.40 ± 0.01	0.39 ± 0.01		0.015
Nonessential amino acids (NEAAs)								
CYS	nd	0.21 ± 0.01	nd	nd	nd	nd		0.004
GLU	1.52 ± 0.22	0.75 ± 0.09	3.22 ± 0.33	3.36 ± 0.31	1.45 ± 0.11	1.56 ± 0.11		-
GLY	0.54 ± 0.06	0.76 ± 0.10	1.65 ± 0.14	1.04 ± 0.09	0.47 ± 0.02	0.63 ± 0.02		-
PRO	0.44 ± 0.02	0.37 ± 0.02	0.97 ± 0.12	3.24 ± 0.24	0.41 ± 0.02	0.55 ± 0.04		-
TYR	0.28 ± 0.01	0.34 ± 0.02	0.63 ± 0.08	0.64 ± 0.04	0.27 ± 0.01	0.31 ± 0.02		Tryo + phyla = 0.025
ALA	0.53 ± 0.95	0.63 ± 0.06	1.32 ± 0.15	1.18 ± 0.11	0.49 ± 0.02	0.58 ± 0.03		-
ASP	0.91 ± 0.1	0.08 ± 0.01	1.88 ± 0.14	2.09 ± 0.21	0.85 ± 0.08	0.93 ± 0.03		-
SER	0.41 ± 0.04	0.66 ± 0.08	0.85 ± 0.09	0.89 ± 0.09	0.35 ± 0.01	0.41 ± 0.02		-
**Amino Acid** **(g/100 g)**	***S. silondia*** *	***S. bacaila***	***C. dussumeri***	***A. chacunda***	***G. telchitta***	***M. cavasius***		**Requirement for Adult Human (g/kg body wt./day)** [12]
**Crude protein (%)**	18.35 ± 0.13	15.05 ± 0.09	18.22 ± 0.08	17.2 ± 0.04	14.9 ± 0.1	16.7 ± 0.01		
Essential amino acids (EAAs)								
ARG	2.00 ± 0.21	0.93 ± 0.04	0.88 ± 0.05	1.00 ± 0.08	0.96 ± 0.08	2.28 ± 018		-
VAL	0.92 ± 0.10	0.48 ± 0.03	0.47 ± 0.02	0.59 ± 0.03	0.53 ± 0.04	1.29 ± 0.09		0.026
HIS	0.46 ± 0.02	0.25 ± 0.01	0.18 ± 0.01	0.34 ± 0.01	0.21 ± 0.02	0.59 ± 0.04		0.01
ILE	0.93 ± 0.10	0.45 ± 0.02	0.43 ± 0.02	0.53 ± 0.04	0.53 ± 0.03	1.33 ± 0.14		0.02
LEU	1.67 ± 0.11	0.85 ± 0.07	0.80 ± 0.05	0.97 ± 0.08	0.95 ± 0.05	2.34 ± 0.23		0.039
LYS	1.66 ± 0.12	0.86 ± 0.07	0.82 ± 0.08	1.04 ± 0.10	0.93 ± 0.05	2.34 ± 0.23		0.030
MET	0.31 ± 0.01	0.16 ± 0.01	0.14 ± 0.01	0.19 ± 0.01	0.13 ± 0.01	0.50 ± 0.04		0.010
PHE	0.83 ± 0.07	0.44 ± 0.02	0.42 ± 0.01	0.52 ± 0.04	0.52 ± 0.02	1.28 ± 0.11		Tryo + phyla = 0.025
THR	0.89 ± 0.07	0.45 ± 0.01	0.40 ± 0.03	0.53 ± 0.04	0.48 ± 0.04	1.20 ± 0.12		0.015
Nonessential amino acids (NEAAs)								
CYS	nd	0.11 ± 0.01	nd	nd	nd	nd		0.004
GLU	0.95 ± 0.09	1.77 ± 0.14	1.66 ± 0.12	1.92 ± 0.18	1.79 ± 0.14	4.62 ± 0.31		-
GLY	0.74 ± 0.04	0.46 ± 0.02	0.49 ± 0.02	0.56 ± 0.02	0.48 ± 0.02	1.08 ± 0.09		-
PRO	0.66 ± 0.04	0.39 ± 0.01	0.39 ± 0.01	0.46 ± 0.02	0.55 ± 0.02	0.98 ± 0.07		-
TYR	0.73 ± 0.06	0.40 ± 0.02	0.36 ± 0.02	0.44 ± 0.02	0.41 ± 0.02	1.00 ± 0.08		Tryo + phyla = 0.025
ALA	1.12 ± 0.11	0.63 ± 0.02	0.57 ± 0.03	0.69 ± 0.05	0.65 ± 0.04	1.55 ± 0.11		-
ASP	0.59 ± 0.03	1.07 ± 0.11	0.96 ± 0.05	1.14 ± 0.11	1.09 ± 0.08	2.77 ± 0.18		-
SER	0.91 ± 0.09	0.44 ± 0.02	0.40 ± 0.02	0.51 ± 0.04	0.47 ± 0.02	1.20 ± 0.02		-
**Amino Acid** **(g/100 g)**	***A. botia***	***E. fusca***	***C. garua***	***C. soborna***		***G. manmina***		**Requirement for Adult Human (g/kg body wt./day)** [12]
**Crude protein (%)**	15.6 ± 0.08	14.5 ± 0.13	18.75 ± 0.10	16 ± 0.09		17.3 ± 0.02		
Essential amino acids (EAAs)								
ARG	2.22 ± 0.11	4.90 ± 0.36	0.58 ± 0.04	0.99 ± 0.11		1.02 ± 0.09		-
VAL	0.97 ± 0.08	3.12 ± 0.34	0.33 ± 0.02	0.47 ± 0.04		0.54 ± 0.02		0.026
HIS	0.43 ± 0.02	1.77 ± 0.021	0.89 ± 0.06	0.24 ± 0.01		0.25 ± 0.03		0.01
ILE	0.87 ± 0.09	3.10 ± 0.24	0.30 ± 0.01	0.42 ± 0.06		0.50 ± 0.06		0.02
LEU	1.53 ± 0.16	5.24 ± 0.41	0.64 ± 0.09	0.75 ± 0.08		0.88 ± 0.09		0.039
LYS	1.46 ± 0.12	4.06 ± 0.33	0.57 ± 0.06	0.74 ± 0.11		0.94 ± 0.06		0.030
MET	0.24 ± 0.01	1.92 ± 0.18	0.14 ± 0.01	0.04 ± 0.01		0.17 ± 0.09		0.010
PHE	0.85 ± 0.10	3.62 ± 0.28	0.34 ± 0.02	0.42 ± 0.06		0.46 ± 0.02		Tryo + phyla = 0.025
THR	0.93 ± 0.10	2.56 ± 0.22	0.37 ± 0.02	0.42 ± 0.06		0.50 ± 0.03		0.015
Nonessential amino acids (NEAAs)								
CYS	0	0.62 ± 0.06	0	0		0		0.004
GLU	3.36 ± 0.22	7.61 ± 0.63	1.38 ± 0.11	1.52 ± 0.08		1.84 ± 0.11		-
GLY	1.04 ± 0.09	3.10 ± 0.28	0.52 ± 0.04	0.54 ± 0.03		0.48 ± 0.03		-
PRO	0.74 ± 0.08	2.35 ± 0.22	0.34 ± 0.02	0.46 ± 0.04		0.38 ± 0.02		-
TYR	0.68 ± 0.07	2.92 ± 0.24	0.24 ± 0.01	0.28 ± 0.01		0.42 ± 0.02		Tryo + phyla = 0.025
ALA	1.25 ± 0.16	3.18 ± 0.32	0.43 ± 0.01	0.53 ± 0.08		0.57 ± 0.05		-
ASP	1.99 ± 0.08	4.55 ± 0.045	0.84 ± 0.06	0.91 ± 0.06		1.04 ± 0.11		-
SER	0.86 ± 0.06	2.51 ± 0.22	0.36 ± 0.01	0.41 ± 0.02		0.47 ± 0.02		-
**Amino Acid** **(g/100 g)**	***C. nama***	***C. latius***	***N. nandus***	***S. gora***	***E. vacha*** *	***C. reba***	***G. giuris***	**Requirement for Adult Human (g/kg body wt./day)** [12]
**Crude protein (%)**	14.99 ± 0.32	13.19 ± 0.07	15.07 ± 0.11	16.8 ± 0.05	19.7 ± 0.08	16.5 ± 0.03	17.5 ± 0.14	
Essential amino acids (EAAs)								
ARG	1.07 ± 0.05	0.13 ± 0.10	0.15 ± 0.01	3.17 ± 0.34	0.64 ± 0.08	1.03 ± 0.04	0.93 ± 0.06	-
VAL	0.69 ± 0.05	0.41 ± 0.02	0.35 ± 0.02	2.14 ± 0.14	0.32 ± 0.03	0.57 ± 0.06	0.48 ± 0.04	0.026
HIS	0.27 ± 0.01	0.95 ± 0.01	0.18 ± 0.02	2.57 ± 0.32	0.15 ± 0.01	0.31 ± 0.02	0.25 ± 0.01	0.01
ILE	0.55 ± 0.07	0.34 ± 0.02	0.01 ± 0.0	2.04 ± 0.14	0.86 ± 0.08	0.53 ± 0.08	0.45 ± 0.03	0.02
LEU	0.96 ± 0.11	0.74 ± 0.07	0.66 ± 0.05	3.36 ± 0.41	0.57 ± 0.05	0.91 ± 0.07	0.81 ± 0. 06	0.039
LYS	1.08 ± 0.09	0.61 ± 0.05	0.80 ± 0.07	2.48 ± 0.18	0.26 ± 0.01	1.26 ± 0.14	1.13 ± 0.11	0.030
MET	0.16 ± 0.01	0.05 ± 0.01	0.15 ± 0.01	1.45 ± 0.11	0.09 ± 0.01	0.16 ± 0.01	0.16 ± 0.01	0.010
PHE	0.50 ± 0.05	0.45 ± 0.04	0.35 ± 0.01	2.13 ± 0.22	0.29 ± 0.01	0.50 ± 0.04	0.46 ± 0.02	Tryo + phyla = 0.025
THR	0.49 ± 0.02	0.64 ± 0.06	0.35 ± 0.01	2.69 ± 0.21	0.30 ± 0.02	0.50 ± 0.04	0.44 ± 0.03	0.015
Nonessential amino acids (NEAAs)								
CYS	nd	nd	nd	0.29 ± 0.02	nd	nd	nd	0.004
GLU	1.97 ± 0.13	1.50 ± 0.11	1.43 ± 0.11	1.63 ± 0.14	1.25 ± 0.14	2.01 ± 0.18	1.81 ± 0.18	-
GLY	0.02 ± 0.0	0.80 ± 0.07	0.37 ± 0.04	0.83 ± 0.10	0.28 ± 0.04	0.51 ± 0.04	0.47 ± 0.03	-
PRO	0.41 ± 0.02	0.43 ± 0.01	0.28 ± 0.02	2.33 ± 0.22	0.24 ± 0.01	1.57 ± 0.14	1.64 ± 0.12	-
TYR	0.44 ± 0.03	0.32 ± 0.01	0.30 ± 0.01	1.81 ± 0.11	0.25 ± 0.04	0.42 ± 0.02	0.37 ± 0.03	Tryo + phyla = 0.025
ALA	0.68 ± 0.05	0.52 ± 0.04	0.50 ± 0.04	2.48 ± 0.25	0.39 ± 0.04	0.69 ± 0.05	0.62 ± 0.04	-
ASP	1.45 ± 0.14	0.92 ± 0.07	0.85 ± 0.05	0.87 ± 0.07	0.75 ± 0.08	1.26 ± 0.11	1.13 ± 0.11	-
SER	0.53 ± 0.02	0.45 ± 0.03	0.36 ± 0.03	2.49 ± 0.22	0.31 ± 0.02	0.51 ± 0.06	0.47 ± 0.02	-

‘nd’ not detected, ‘*’ large fish, ‘^†^’ shellfish. Values are expressed as means ± standard deviations (*n* = 6).

**Table 2:** Fish species rich in different amino acids among the 30 food fish studied.

Essential amino acids (EAAs)					
ARG ^c^	*E. fusca*	*S. gora*	*M. cavasius*	*A. botia*	*S. silondia*
VAL	*E. fusca*	*M. malcomsonii*	*M. cavasius*	*C. cynoglossus*	*A. botia*
HIS	*M. malcomsonii*	*E. fusca*	*C. morar*	*S. ruconius*	*A. bato*
ILE	*E. fusca*	*M. malcomsonii*	*M. cavasius*	*C. cynoglossus*	*S. silondia*
LEU ^c^	*E. fusca*	*M. malcomsonii*	*M. cavasius*	*S. silondia*	*C. cynoglossus*
LYS	*E. fusca*	*M. cavasius*	*M. malcomsonii*	*C. cynoglossus*	*S. silondia*
MET ^c^	*E. fusca*	*S. gora*	*M. malcomsonii*	*M. cavasius*	*C. cynoglossus*
PHE	*E. fusca*	*M. malcomsonii*	*M. cavasius*	*C. cynoglossus*	*A. botia*
THR	*M. malcomsonii*	*E. fusca*	*M. cavasius*	*A. botia*	*S. silondia*
Nonessential amino acids (NEAAs)					
CYS	*G. cenia*	*E. fusca*	*A. bato*	*S. bacaila*	
GLU ^ac^	*E. fusca*	*M. cavasius*	*A. botia*	*C. cynoglossus*	*M. panculus*
GLY ^ac^	*E. fusca*	*M. malcomsonii*	*M. panculus*	*M. cavasius*	*C. cynoglossus*
PRO ^ac^	*C. cynoglossus*	*E. fusca*	*S. gora*	*G. giuris*	*C. reba*
TYR ^c^	*M. malcomsonii*	*E. fusca*	*M. cavasius*	*S. silondia*	*A. botia*
ALA	*E. fusca*	*S. gora*	*M. malcomsonii*	*M. cavasius*	*M. panculus*
ASP	*E. fusca*	*M. cavasius*	*C. cynoglossus*	*A. botia*	*M. panculus*
SER	*E. fusca*	*S. gora*	*M. malcomsonii*	*M. cavasius*	*C. morar*

^a^: Conditionally essential amino acids; ^c^: functional amino acids as per human nutrition (Wu, 2010) [3].

## Data Availability

The original contributions presented in the study are included in the article, further inquiries can be directed to the corresponding author.
